# Gender‐stereotypical attribution of fear and fearlessness in preschool children

**DOI:** 10.1111/bjdp.12539

**Published:** 2024-12-18

**Authors:** Sofia Kelesidou, Fotini Bonoti, Georgios Abakoumkin, Plousia Misailidi

**Affiliations:** ^1^ Laboratory of Psychology, Department of Early Childhood Education University of Thessaly Volos Greece; ^2^ Department of Nutrition and Dietetics University of Thessaly Trikala Greece; ^3^ Department of Primary Education University of Ioannina Ioannina Greece

**Keywords:** emotion stereotypes, fear, fearlessness, gender, preschool children, stereotypes

## Abstract

Fear and fearlessness are two distinct emotional responses that can occur when a person faces a potentially dangerous situation. These responses seem to be linked to gender stereotypes (fearful female and fearless male). This study examines whether preschool children attribute fear and fearlessness stereotypically in relation to gender and whether their attributions vary as a function of age and gender. One hundred and twenty children (60 boys and 60 girls) aged 3, 4 and 5 years participated in this study. We examined children's gender‐stereotypical performance through emotional scenarios and drawing tasks involving fear and fearlessness conditions. The results showed that children's performance was equally stereotypical in the two conditions. An age effect was found: children's gender stereotypical attributions increased with age. The results showed no differences in children's stereotypical performance in regards to their gender and task type (emotional scenarios and drawing tasks). The significance and implications of these findings are discussed in the context of gender‐emotion stereotypes.

## Graphical abstract

[Correction added on 14 June 2025: Graphical Abstract has been included in this version.]

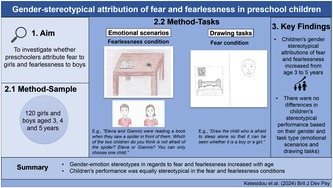

**Key points**

- Preschool children stereotypically attribute fear to female protagonists and fearlessness to male protagonists in both emotional scenarios and drawing tasks, reflecting societal norms of femininity and masculinity.
- The study found that gender‐stereotypical attributions of fear and fearlessness increase with age during the preschool years (ages 3–5).
- Boys and girls displayed similar levels of gender‐stereotypical performance in both fear and fearlessness conditions, suggesting that these gender‐emotion stereotypes are equally internalized during early childhood.

## INTRODUCTION

Gender affects how we perceive emotions, both in others and within ourselves (Shields et al., 2018). One of the most prevalent beliefs regarding gender differences is that women are often considered more emotional than men (Grossman & Wood, 1993; Shields, 2013). Women also exhibit greater emotional expressivity than men, particularly in the context of negative emotions such as horror and disgust (Deng et al., 2016). The socialization of men and women imparts different goals and motives, influenced by age and cultural background, thereby leading to distinct emotional processes (Brody & Hall, 2008). In addition, women differ from men in their emotional experiences, as emotions such as fear and sadness seem to play a more prominent role in women's lives, whereas emotions such as anger and disgust seem to have a greater significance in men's lives (Schirmer, 2013).

To understand how these gender‐based differences in emotional expression and perception develop, it is important to examine the role of gender stereotypes in shaping individual behaviour and societal expectations. Gender stereotypes are traits or characteristics commonly associated with boys or girls as a group. While these traits can be viewed as positive or negative, stereotypes tend to amplify differences between groups (Brown & Stone, 2016). These stereotypes emerge during childhood and are encouraged both by society and the children's family environment. Τhe emergence of gender stereotypical behaviours is considered an indicator of children's increasing understanding of gender stability during the preschool years (Halim & Ruble, 2010). Gender stereotypes pervade various aspects of children's lives extending from cartoons (Baker & Raney, 2007; Tzoutzou et al., 2021) to books (Fitzpatrick & McPherson, 2010; Seitz et al., 2020). Children's acceptance of gender stereotypes significantly shapes how they perceive themselves and can potentially affect their future career choices (Master et al., 2021; Vivaldi & Rose, 2024). While studies on gender stereotypes have increased in the last decades, more insight is needed into how various factors shape these stereotypes and how they affect children's development (de Morais et al., 2024).

In the present study, we examined whether preschool children stereotypically attribute fear and fearlessness to male and female gender and whether their performance differs by age and gender.

### Emotion stereotypes—fear and fearlessness

Emotions serve an adaptive function, aiding individuals to achieve both intrapersonal and interpersonal goals (Brody & Hall, 2008). Given the divergent goals arising from the socialization of men and women, it is anticipated that the emotional expression will also vary by gender (Brody & Hall, 2008). A main position of Eagly's (1987) Social Roles Theory is that gender roles are based on agentic/communal characteristics. Specifically, it is argued that men's roles are centred on agency (e.g. being courageous, having heroic behaviour, being aggressive, strong, leading, dominating, assertive, competitive, etc.), whereas women's roles are rooted in communality (e.g. being caring, sensitive, friendly, emotional, nurturing, etc.) (Eagly et al., 2003; Eagly et al., 2020; Eagly & Johannesen‐Schmidt, 2001).

Fear and fearlessness can be viewed as distinct emotional states or traits that may be elicited by similar stimuli or situations. Fear is an emotion that arises in response to threatening situations involving a risk of physical and social harm (Schirmer, 2013). It is a fundamental emotion that plays a critical role in survival by mobilizing an organism's resources in the face of danger (Adolphs, 2013). Fear as a state is an adaptation to a specific situation (e.g. freezing response) and is different from trait fear which is a consistent characteristic of a person across time and situations (Sylvers et al., 2011). Fearlessness, on the other hand, can be understood as low sensitivity in situations where a fearful response would typically be expected, along with a limited physiological response to aversive or threatening stimuli (Fanti et al., 2023).

Research indicates that women tend to report experiencing fear more intensely than men (McLean & Anderson, 2009). The meta‐analysis by Chaplin and Aldao (2013) examined emotional expression in infants, children and adolescents (0–17 years) and reported that girls expressed more fear than boys, especially in negative situations or in the presence of an unfamiliar adult. In another meta‐analysis of gender differences in temperament in children aged from 3 months to 13 years, girls showed higher fearfulness than boys (Else‐Quest et al., 2006). This tendency may be attributed to gender stereotypes, since it has been argued that the emotion of fear is considered more acceptable when expressed by the female gender, whereas boys are encouraged to display more confidence and reduce the levels of fear they express (Ginsburg & Silverman, 2000).

It has been argued that gender stereotypes regarding fear lead boys to deny their vulnerability and exhibit fearlessness or reactive aggression towards others (Goodey, 1997). Women are raised to be more fearful while men are encouraged to be fearless due to the impact of socialization processes (Cops & Pleysier, 2011). This tendency appears to manifest in preschool age. In Albert and Porter's (1983) study, children aged 4–6 years were asked to attribute gender‐role stereotypical traits to a male and a female doll, and the results revealed that both boys and girls did so accordingly (e.g. associating traits like fearlessness with the male doll and nonaggression with the female doll). In a cross‐cultural study exploring young adults' desires for their future children's emotions, the two most desirable emotions were happiness and fearlessness. The desire for fearlessness was more pronounced in male participants and was considered a more desirable emotion for sons, but less so for daughters (Diener & Lucas, 2004).

Research has also highlighted that overcoming fear is one of the characteristics that describe courage (Rate et al., 2007), which according to Eagly's (1987) Social Role Theory is a personality trait that could have stereotypical implications, as it is an agentic characteristic attributed to males. As early as 5 years, children perceive lack of fear as one of the dimensions of courage (Bonoti, 2018; Szagun, 1992). Heroes are often depicted as fearless (Hansen, 2018) and courageous (Kinsella et al., 2017), traits that are agentic and frequently associated with men (Eagly et al., 2020).

While research suggests that women experience higher levels of fear than men, evidence regarding biological differences between genders in relation to fear is not definitive (Kinsella et al., 2017). Evolutionary theory suggests that men evolved to take risks and exhibit lower fear levels due to mating competition, enhancing reproductive success despite dangers. On the other hand, the heightened fear sensitivity in women is seen as an adaptive response for personal survival and the protection of their offspring (Campbell et al., 2021). Socialization theories, such as Social Role Theory (Eagly & Wood, 2012), suggest that gender role beliefs are encouraging gender conforming behaviours and traits in men and women. For example, fearful behaviour might be less acceptable in boys (McLean & Anderson, 2009). Family and cultural dynamics may prompt men to either suppress or downplay their feelings of fear, to reinterpret moderate fear levels as pleasant, or even to endure uncomfortable levels of fear to uphold a ‘masculine’ facade (Campbell et al., 2021). Perceptions about bravery and, consequently, fearlessness may also be influenced by gender socialization (Kinsella et al., 2017). Stereotypes of ‘fearless males’ are believed to be particularly detrimental for boys, as they may refrain from expressing vulnerability or fear to avoid compromising their masculine identity (Goodey, 1997). Moreover, these stereotypes can have a negative impact on women, reducing the likelihood of them behaving without fear in certain situations. Similarly, they may impact men who might feel pressure to act in potentially dangerous scenarios (Kinsella et al., 2017).

### The present study

The aim of this study was to investigate whether preschool‐aged children attribute fear and fearlessness stereotypically in relation to gender and whether their performance varies as a function of age and gender. This study combines fear and fearlessness tasks, as we attempt to examine whether children's performance will exhibit similarities or differences across the two conditions. To examine children's stereotypical performance in regards to the emotion of fear, we implemented emotional scenarios and drawing tasks. Adopting the methodological approach of prior research (as documented in Brechet, 2013, and Brechet et al., 2009), we seek to investigate the hypotheses by employing two distinct task types, namely emotional scenarios and drawing tasks. Both tasks are commonly used in emotion research, predominantly due to their suitability for exploring emotions in young children (Brechet, 2013; Brechet et al., 2009; Parhomenko, 2014; Siedlecka & Denson, 2019). Emotional scenarios consist of hypothetical stories in which children attribute emotions to the protagonists, revolving around emotional situations (Bretherton et al., 1986). Drawing tasks are also an important tool in preschool research because young children may not possess the ability to express their beliefs and emotions verbally (Antona, 2019).

In the fear condition, we asked children ‘which child do you think is afraid of…’ and ‘draw me the child who is afraid of…’, whereas in the fearlessness condition, the instructions were reversed using negative sentences. For example, the sentence ‘which child do you think is afraid?’ became ‘which child do you think is not afraid?’ and the instruction ‘draw me the child who is afraid…’ became ‘draw me the child who is not afraid…’. The ‘negative’ instructions given are developmentally appropriate for the ages of the children being examined. Children can understand and use ‘no’ and ‘not’ after 2 years of age (Feiman et al., 2017) and 3‐year‐olds understand negative sentences faster and more successfully compared to younger children (Nordmeyer & Frank, 2013). In this study, we selected fears from the FSSC‐R scale (Ollendick, 2006) that are developmentally appropriate and commonly observed in preschool‐aged children. Specifically, we chose the fear categories ‘Small animals’ and ‘The unknown’, as research has shown that fears related to animals and the unknown are prevalent during early childhood (Burnham & Gullone, 1997; Coelho et al., 2021; Loxton, 2009; Mellon et al., 2004; Muris & Merckelbach, 2001). For the emotional scenarios, we chose (a) a spider and a worm, representing the fear category ‘Small animals’ and (b) dark room and sea, representing the fear category ‘The unknown’. For the drawing tasks, we selected (a) a mouse and a lizard representing the fear category ‘Small animals’ and (b) a child sleeping alone and a dark forest, representing the fear category ‘The unknown’. In total, the emotional scenarios and drawing tasks included these eight fear items to ensure a representative sample of fears commonly experienced during the preschool years.

Our research was based on Eagly's (1987) Social Role Theory, which states that different roles and behaviours are expected of men and women in society. According to this theory, men are expected to exhibit agentic characteristics (e.g. assertiveness, bravery, confidence), whereas women are expected to exhibit communal characteristics (e.g. nurturance, kindness, sensitivity). These different social roles shape individuals' behaviour, including how emotions are expressed and perceived (Eagly, 1987; Eagly & Wood, 1991). In the context of our study, Social Role Theory helps explain how gender‐emotion stereotypes—such as the expectation that women are more likely to express fear, whereas men are expected to appear fearless— are internalized from an early age. Because of these socially defined roles, we hypothesize that children's performance in the emotional scenarios and drawing tasks will be influenced by gendered expectations about fear and fearlessness. Note that we acknowledge the diversity of the gender spectrum and recognize that categorizing it within the confines of a binary framework, such as female/male or boy/girl, is restrictive. While acknowledging that gender is not binary, our research focuses specifically on girls and boys to highlight how traditional, binary expectations regarding gender and emotional responses influence children's performance. The decision to maintain a binary framework for gender categorization was driven by the specific focus on preschool children, as young as 3 years old, and the current limitations in empirical evidence regarding their awareness and understanding of diverse gender identities. At this developmental stage, children are beginning to grasp the concept of gender, but their comprehension is often rooted in the most visible and traditionally recognized categories, which are male and female (Martin & Ruble, 2010). Based on this theoretical position and other relevant research data, we hypothesize the following:

Hypothesis 1 *Based on Eagly's (* 1987 *) Social Role Theory, fearlessness could be considered a characteristic of the male role (achievement‐agentic) and fear a characteristic of the female role (communal). Therefore, we anticipated that children's performance would be stereotypical in both fear and fearlessness conditions in the emotional scenarios and drawing tasks. As children are familiar with gender roles, we expected that they would attribute the condition of fear more often to female protagonists and the condition of fearlessness to male protagonists*.

Hypothesis 2 *As stereotypes increase with age and peak around 6 years (Trautner* et al., 2005 *), we expected that older children would have higher stereotypical performance than younger ones*.

Hypothesis 3 *Because fear and fearlessness are associated with females and males respectively (Goodey*, 1997; *Widen & Russel*, 2002 *) and preschool children have access mainly to stereotypes related to their gender (Martin*, 2000 *), we expected that girls would have a higher stereotypical performance in the fear condition and boys in the fearlessness condition*.

The research design of the present study was 2 (participant gender: boys and girls) × 3 (age: 3 years, 4 years and 5 years) × 2 (type of task: emotional scenario tasks and drawing tasks) × 2 (conditions: fear–fearlessness) with repeated measures on the last two factors.

## METHOD

### Sample

Participants were 120 children (60 boys and 60 girls) aged 3 (*n* = 40, *M* = 41.02 months, *SD* = 3.80), 4 (*n* = 40, *M* = 54.05 months, *SD* = 3.36) and 5 years (*n* = 40, *M* = 66.22 months, *SD* = 3.03) attending kindergartens and day care centres in the region of Attica, Greece. The research was approved by the Greek Ministry of Education (Protocol No. F15/186706/219134/D1). Additionally, we distributed a consent form to the parents of the children. Parents who informed us that their children exhibited phobias were advised against providing consent for these children's participation in the study.

### Procedure

#### Preparatory task

We conducted a preparatory task in order to ensure that children were able to distinguish between fear and fearlessness. Half of the children started with the fear condition and the other half with the fearlessness condition.

**FIGURE 1:**
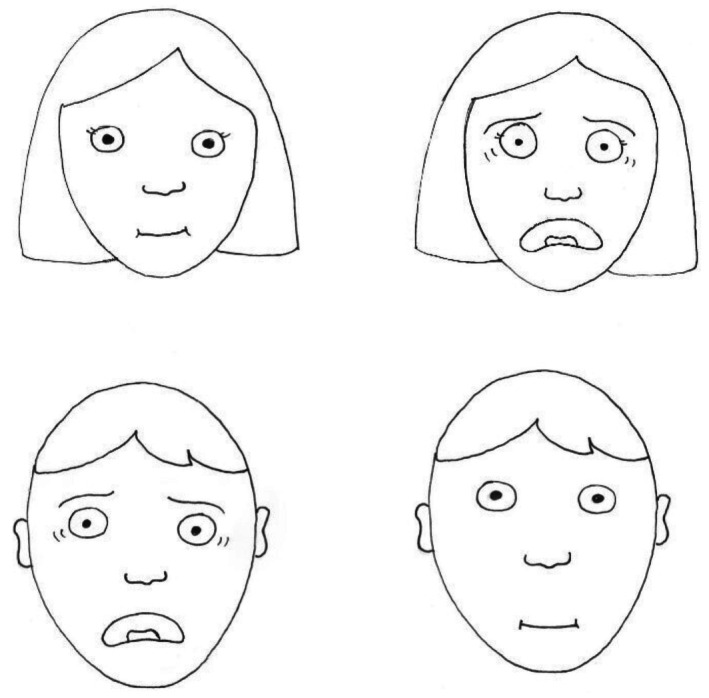
Cards with fearful and neutral expressions.

Specifically, we presented four cards to the children, demonstrating the faces of two boys and two girls with fearful and neutral expressions. The design of the faces was based on the illustrations of the fear scale from Sayfan and Lagattuta (2009) (Figure 1). We presented the cards in pairs, starting from either the boys' or the girls' faces. In one of the two pairs, we asked children to indicate the fearful face (‘Show me the boy/girl who is afraid’). In the other pairs, we asked them to indicate the neutral face (‘Show me the boy/girl who is not afraid’). The pairs of faces were then alternated. Children who failed to answer correctly (*n* = 2) were excluded from the main study.

### Emotional scenarios

**FIGURE 2:**
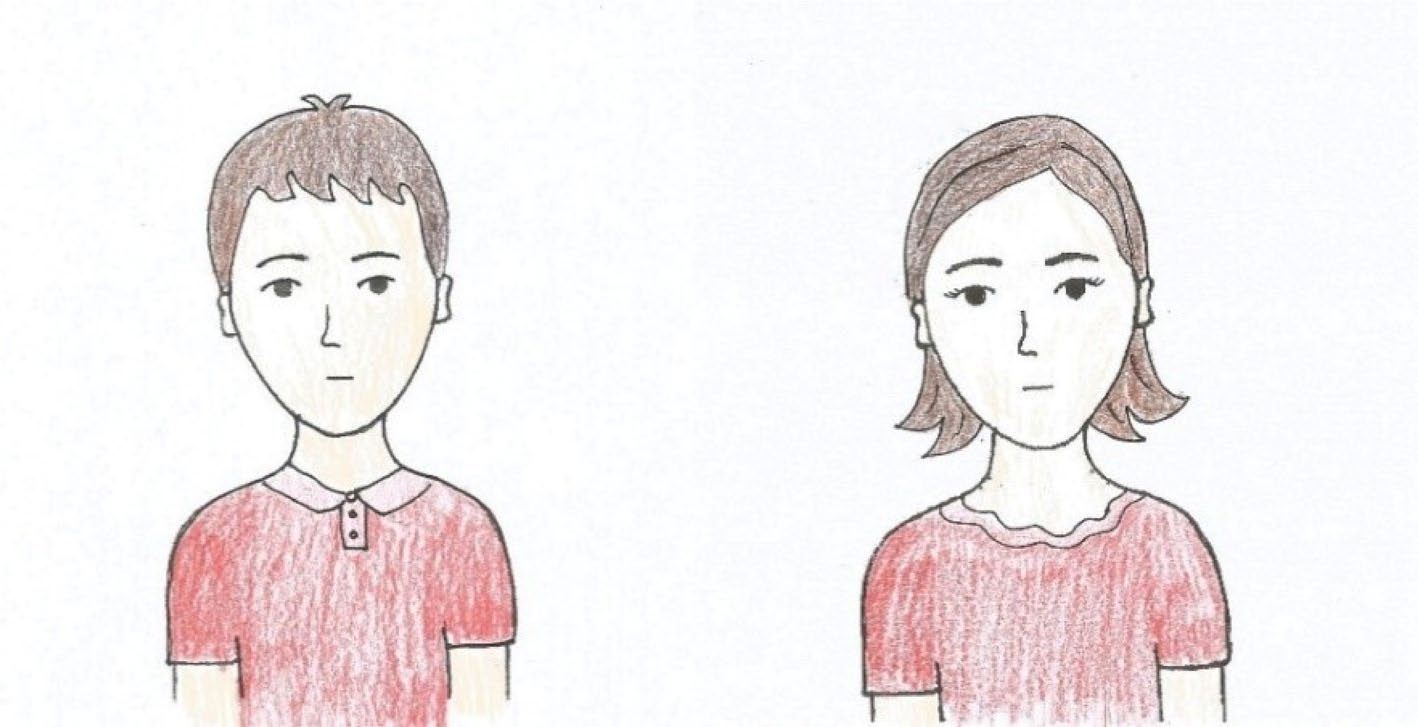
Example of boy and girl with emotionally neutral expression.

The emotional scenarios were accompanied by pictures to help children visualize more easily the protagonists and the fearful stimuli presented. Specifically, we designed four different pairs of protagonists (boy and girl) (Figure 2), such that the children would not be influenced by the answers they gave about the protagonists in each scenario.

#### Emotional scenarios administration

We tested all children in both conditions (fear/fearlessness). To avoid influencing children's attributions by using the same items in fear and fearlessness conditions and thus eliciting opposite answers for each one, we decided to use different fear items from the selected fear categories (small animal fears and fears of the unknown) for the two conditions. Two pairs of fear items were utilized to test children's gender stereotypical attributions in the conditions of fear and fearlessness in the emotional scenarios (first pair: spider‐dark room, second pair: worm‐sea) (see Table 1). The children were divided into two groups. Specifically, the fear items used in the fear condition in the first group were employed in the fearlessness condition in the second group, and vice versa, as indicated in Table 1. We employed this strategy for balancing purposes so that the content of the fear items would not affect children's performance in the fear and fearlessness conditions.

**TABLE 1:** Images and instructions of emotional scenarios.

Emotional scenarios	Fear condition	Fearlessness condition
First group	(1) Elena and Giannis were reading a book when they saw a spider in front of them. Which child of the two do you think is afraid of the spider? Elena or Giannis? You can only choose one child. 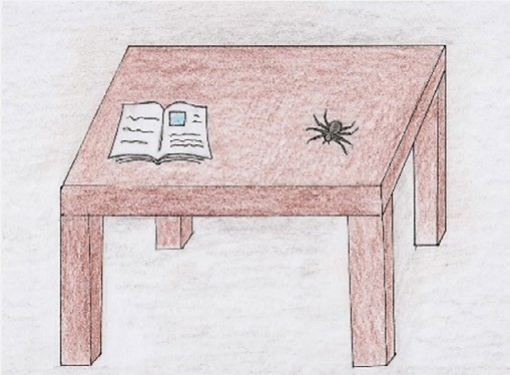	(1) Pavlos and Stella were playing in the yard when they saw a worm in the dirt. Which child of the two do you think is not afraid of the worm? Pavlos or Stella? You can only choose one child. 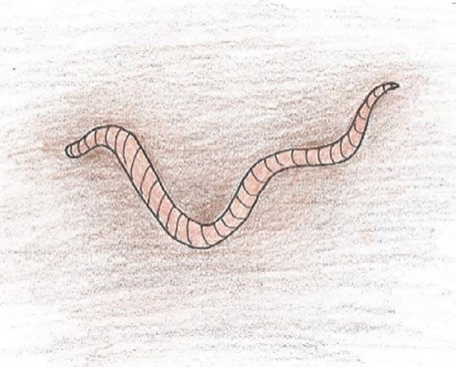
	(2) It is bedtime. Which child do you think is afraid to sleep in the dark? Nickos or Eirini? You can only choose one child. 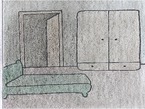	(2) Katerina and Andreas went for a walk by the sea. There were a lot of waves. Which of the two children do you think is not afraid of the waves? Katerina or Andreas? You can only choose one child. 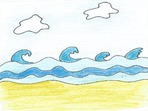
Second group	(1) Pavlos and Stella were playing in the yard when they saw a worm in the dirt. Which child do you think is afraid of the worm? Pavlos or Stella? You can only choose one child. 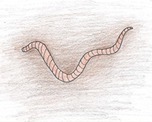	(1) Elena and Giannis were reading a book when they saw a spider in front of them. Which of the two children do you think is not afraid of the spider? Elena or Giannis? You can only choose one child. 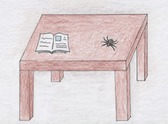
	(2) Katerina and Andreas went for a walk by the sea. There were a lot of waves. Which of the two children do you think is afraid of the waves? Katerina or Andreas? You can only choose one child. 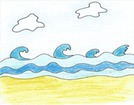	(2) It is bedtime. Which child do you think is not afraid to sleep in the dark? Nickos or Eirini? You can only choose one child. 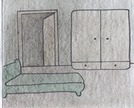

The same procedure was followed for both the fear and fearlessness conditions. In both conditions, we presented children with four emotional scenarios in a random order (the sequence of scenarios for each child was pre‐determined using a random number generator website). Initially, we displayed the pictures of one pair (out of four pairs) of protagonists and told the children their names. Subsequently, we presented to the children the emotional scenarios, each depicted in a single picture. Then we asked them to indicate which protagonist, boy or girl, was frightened or not afraid by the situation described in the scenario (e.g. spider, darkness). We emphasized that they could choose only one of the two protagonists. Correspondingly, the fearlessness condition followed the same structure but with a focus on identifying fearlessness. The exact instructions are shown in Table 1.

### Drawing tasks

In the drawing tasks, we provided children with pencils and two sheets of paper, each sheet containing a fear item in a random order. In the fear condition, a pre‐drawn human face with a fearful expression accompanied each fear item, whereas in the fearlessness condition, a pre‐drawn human face with a neutral expression was presented together with each item. We intentionally designed both faces to be genderless, as shown in Table 2. Recognizing that children often use cues like hair length and clothing to determine gender (Cox, 2005), we provided extra space under each face for children to add elements such as a body or clothes if they chose. In the fear condition, we asked the children to ‘draw the child who is afraid so that it can be seen whether it is a girl or a boy (*or* a boy or girl)’. In the fearlessness condition, we asked children to ‘draw the child who is not afraid so that it can be seen whether it is a boy or a girl (*or* girl or a boy)’. The order of gender (boy/girl or girl/boy) in the questions was alternated for balancing purposes. With this task we aimed to elicit the children's perceptions of gender in the context of fear and fearlessness.

**TABLE 2:** Images and instructions of drawing tasks.

Drawing tasks	Fear condition	Fearlessness condition
First group	(1) Draw the child who is afraid to sleep alone so that it can be seen whether it is a boy or a girl (*or* a girl or a boy). 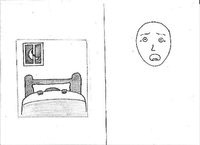	(1) Draw the child who is not afraid to walk in the dark forest, so that it can be seen whether it is a boy or a girl (*or* a girl or a boy). 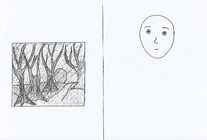
	(2) Draw the child who is afraid of the mouse, so that it can be seen whether it is a girl or a boy (*or* a boy or a girl). 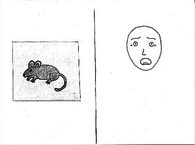	(2) Draw the child who is not afraid of the lizard, so that it can be seen whether it is a girl or a boy (*or* a boy or a girl). 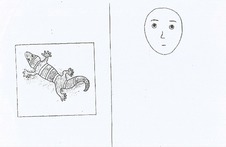
Second group	(1) Draw the child who is afraid to walk in the dark forest, so that it can be seen whether it is a boy or a girl (*or* a girl or a boy). 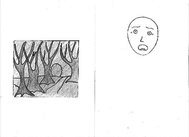	(1) Draw the child who is not afraid to sleep alone, so that it can be seen whether it is a boy or a girl (*or* a girl or a boy). 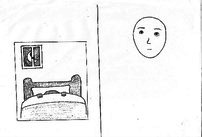
	(2) Draw the child who is afraid of the lizard, so that it can be seen whether it is a girl or a boy (*or* a boy or a girl). 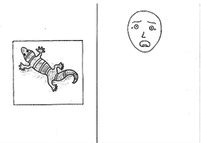	(2) Draw the child who is not afraid of the mouse, so that it can be seen whether it is a girl or a boy (*or* a boy or a girl). 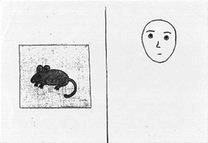

In both the fear and fearlessness drawing tasks, our primary objective was to explore how children associate gender with different emotional states—fear and fearlessness—through their drawings. The consistent use of non‐gendered faces beside the fear items ensured that any gendered characteristics added in the drawings stemmed from the children's own perceptions and interpretations.

### Scoring

Each time the children selected the girl or drew a girl in the emotional scenarios and drawing tasks, respectively, for the fear condition, we considered their performance as stereotypical and their scoring was 1 point. Conversely, each time they selected the boy or drew a boy, we considered their performance as non‐stereotypical and their scoring was 0 points. Similarly, each time the boy was selected or drawn in the emotional scenarios and drawing tasks, respectively, for the fearlessness condition, we considered their performance as stereotypical, and their scoring was 1 point. Conversely, each time the girl was selected or drawn, we considered their performance as non‐stereotypical, and their scoring was 0 points. Then, we assigned each child a total stereotypical performance score for the fear condition and a total stereotypical performance score for the fearlessness condition (with a score range from 0 to 2 points for each condition).

## RESULTS

The design of the present study was 2 (participant gender: boys and girls) × 3 (age: 3 years, 4 years and 5 years) × 2 (type of task: emotional scenario tasks and drawing tasks) × 2 (conditions: fear–fearlessness) with repeated measures on the last two factors. The dependent variable is the stereotypicality of fear. We conducted a preliminary analysis using analysis of variance (ANOVA), with the order in which the children were administered the tasks as the independent variable, and the children's performance in the emotional scenarios and drawing tasks as the dependent variable. This analysis did not reveal statistically significant differences in children's performance [Emotional scenarios/fear *F*(1,118) = .024, *p* = .878 Emotional scenarios/fearlessness *F*(1,118) = .003, *p* = .96. Drawing tasks/fear *F*(1,118) = .717, *p* = .399. Drawing tasks/fearlessness *F*(1,118) = 1.86, *p* = .18]. Therefore, we did not include the order of the task presentation in the subsequent analyses. The results for each hypothesis are presented below.

The findings of the study support Hypothesis 1, as children's stereotypical performance did not differ between the two conditions (fear and fearlessness) in both emotional scenarios and drawing tasks *F*(1,114) = .08, *p =* .78. Specifically, the mean stereotypical performance in the fear condition (*M* = 1.13, *SD* = .37) and the fearlessness condition (*M* = 1.14, *SD* = .40) were similar.

The analysis also indicated a statistically significant main effect of age *F*(2,114) = 3.37, *p* = .04, *η* ^*2*^ = .06, supporting Hypothesis 2. As shown in Table 3, children's stereotypical performance in both emotional scenarios and drawing tasks increased with age. Specifically, the mean scores related to stereotypical performance of the 3‐year‐old children (*M* = 1.06, *SD* = .18) are lower than the mean scores of the 4‐year‐old children (*M* = 1.13, *SD* = .31), while the highest stereotypical performance is seen in the 5‐year‐old children (*M* = 1.20, *SD* = .22). As can be seen, the mean stereotypical performance of the 3‐year‐old children is the lowest among the three age groups and is above the midpoint of the rating scale (0–2 points). Post hoc comparisons of means using Tukey's test showed statistically significant differences only between the 3‐year‐old and the 5‐year‐old children (*p* < .05).

**TABLE 3:** Means and standard deviations of stereotypical performance by age group and type of task.

Age groups	Type of task		Total
	Emotional scenarios	Drawing tasks	
3‐year‐olds	1.02 (.26)	1.10 (.26)	1.06 (.18)
4‐years‐olds	1.12 (.39)	1.14 (.33)	1.13 (.31)
5‐years‐olds	1.18 (.32)	1.22 (.30)	1.20 (.22)

Hypothesis 3 is not supported by the data obtained, as the effect of children's gender was not found to be statistically significant *F*(1,114) = .186, *p =* .67 and did not appear to differentiate their stereotypical performance. The means of stereotypical performance of boys (*M* = 1.14, *SD* = .30) and girls (*M* = 1.12, *SD* = .34) were similar.

The findings regarding the effect of task type (emotional scenarios and drawing tasks) showed that children's performance in the two tasks did not differ significantly, *F*(1,114) = .41, *p* = .52. The mean stereotypical performance in the emotional scenarios (*M* = 1.11, *SD* = .32) was only slightly lower than the mean in the drawing tasks (*M* = 1.15, *SD* = .30). Notably, there were no further main effects or interactions that were statistically significant.

## DISCUSSION

The aim of the present study was to examine whether children aged 3–5 years would stereotypically attribute fear and fearlessness to protagonists in emotional scenarios and drawing tasks, and whether their stereotypical performance would vary as a function of age and gender. Τhe findings of the study are presented by hypothesis and their significance is discussed.

As hypothesized, children's performance was equally stereotypical in the fear and fearlessness conditions, as they attributed fear more often to girls and fearlessness more often to boys, which suggests the early internalization of gender norms, consistent with societal expectations of masculinity and femininity (Chaplin, 2015). These findings support Hypothesis 1, confirming that preschool children exhibit gender‐stereotypical perceptions of fear and fearlessness. These results are consistent with previous studies showing that the emotion of fear is more often attributed to females (Capriola et al., 2017; Ginsburg & Silverman, 2000; Parmley & Cunningham, 2007), while fearlessness is more often attributed to males (Albert & Porter, 1983). These findings align with Eagly's (1987) Social Role Theory, where it is argued that men's roles are agentic (e.g. being courageous, having heroic behaviour, being aggressive, strong, leading, etc.), whereas women's roles are communal (e.g., being caring, sensitive, friendly, emotional, etc.) (Eagly et al., 2003; Eagly et al., 2020; Eagly & Johannesen‐Schmidt, 2001; Rankin & Eagly, 2008).

Consistent with Hypothesis 2, an age‐related effect was observed with 5‐year‐old children showing a higher stereotypical performance than 3‐year‐old children. Our findings, consistent with previous research showing that gender stereotypes in early childhood increase between 3 and 5 years (Campbell et al., 2004; Halim & Ruble, 2010; Miller et al., 2009), suggest that children's stereotypical performance regarding the emotion of fear increases with age, likely due to their increasing exposure to gender‐emotion stereotypes in their environment (parental reinforcing, books, television, etc.). It has been argued that at 4 years of age, gender knowledge increases rapidly, and children can demonstrate their knowledge due to their developing cognitive and language skills (Leinbach et al., 1997). As children grow and engage more with their peers, they absorb and mimic behaviours observed in their social environment. Peer interactions can reinforce gender stereotypes through shared play activities, conversations and the modelling of gendered behaviours. Gender stereotypes, revolving around male dominance/assertiveness and female passiveness, can significantly influence children's perceptions and reactions to various peer behaviours (Doey et al., 2014).

Contrary to Hypothesis 3, no significant gender differences were found in children's gender stereotypical performance when attributing fear and fearlessness. This suggests that preschool boys and girls are equally receptive to gender‐emotion stereotypes regarding fear and fearlessness. It is likely that the reported gender effects may emerge at older ages when children are acquainted with specific fears (Gullone, 2000; Muris et al., 2000; Talu, 2019) as opposed to the preschool years, which constitute the primary focus of the current study. In the preschool years, children are still developing their emotional literacy, which includes recognizing and categorizing emotions in themselves and others (Harper, 2016). As they grow older and their cognitive and emotional abilities mature, their understanding of fear and societal expectations concerning its expression might become more gendered.

Task type (emotional scenarios and drawing tasks) had no effect on children's stereotypical performance when attributing fear and fearlessness to male and female protagonists. It should be noted, however, that the drawing tasks were very simple, requiring the children to depict only the gender of the child and not her or his emotion which was already depicted. We made this decision because the accurate graphic depiction of fear can be challenging for children under 5 years (Cox, 2005). In contrast, children as young as 3 years can represent gender in their drawings using elements like hair (Golomb, 2011). The findings support the conclusion that both emotional scenarios and drawing tasks can be used equally effectively in studies involving children aimed at investigating the understanding of emotions (Brechet et al., 2009). Consequently, it seems that they can act as complementary tools in preschool age to investigate gender‐emotion stereotypes (Brechet, 2013), always bearing in mind that they should be designed to be as simple as possible considering the young ages targeted.

### Limitations and future research directions

The researcher's gender (female) is a factor that could have influenced the children's stereotypical performance (Halim & Ruble, 2010; Parmley & Cunningham, 2007), taking also into account that children might alter the content of their emotion‐related drawings depending on the gender of the audience (Brechet et al., 2021). Furthermore, due to the design of the research, children could only choose the boy or the girl in each condition (fear and fearlessness) in the emotional scenarios and drawing tasks, which is a limitation as they were not given the opportunity to choose both genders. Additionally, the pictures of the faces of the protagonists accompanying the emotional scenarios had neutral expressions which could affect children's judgements in the condition of fear, as it could possibly make it difficult for them to understand the emotion experienced by the protagonists. In the condition of fearlessness, the neutral expression of both protagonists may also influence children's judgements by considering that neither protagonist experiences fear. Furthermore, in order to balance tasks, we divided the children into two groups to ensure that the content of the fear items did not impact their performance in the fear and fearlessness conditions. Consequently, although the fear items were drawn from the same fear categories and were administered inversely to the two groups to balance the fear and fearlessness conditions, this could have influenced children's performance in the two groups, as they were not tested on exactly the same items in both conditions. Finally, the fear items we tested were limited due to the age of the children and it is possible that different performance would occur if different items were chosen.

Future research should explore whether differences exist between fear and fearlessness across a broader range of fear‐related items than those used in the present study and also focus on older age groups. Additionally, an exclusive focus on the study of fearlessness could provide a more detailed examination of the development of gender stereotypes concerning fearlessness in preschool children, an area that has not been extensively explored.

## CONCLUSION

Despite the positive changes in the landscape of gender stereotypes in recent years, challenges still remain. The current study showed that children's performance was equally stereotypical in the fear and fearlessness conditions. Moreover, the findings show that gender‐emotion stereotypes in regards to fear and fearlessness increase from age 3 to 5 years, consistent with previous research on stereotypes showing a similar developmental pattern (Campbell et al., 2004; Halim & Ruble, 2010; Miller et al., 2009; Ruble et al., 2007).

Children's exposure to gender stereotypes negatively impacts their social and emotional development by limiting the range of acceptable characteristics they feel they can express (Adams et al., 2011), thus leading to a constrained exploration of interests, emotions and activities. Specifically, children learn gender stereotypes and the gender roles they should adopt through the expectations of their parents, teachers and other social influences such as the media (Wigfield et al., 2015). When widespread beliefs influence individuals' emotional responses and behaviours, sexism may manifest as gender‐based prejudice (Brown, 2017). This perpetuates gendered behaviours that can be harmful to boys and girls, as it restricts their ability to explore a full range of emotions and interests.

## AUTHOR CONTRIBUTIONS

**Sofia Kelesidou:** Conceptualization; investigation; writing – original draft; methodology; writing – review and editing; data curation; formal analysis. **Fotini Bonoti:** Methodology; writing – review and editing; conceptualization; supervision. **Georgios Abakoumkin:** Methodology; conceptualization. **Plousia Misailidi:** Conceptualization; methodology.

## CONFLICTS OF INTEREST

The authors declare no conflicts of interest.

## Data Availability

Data and Materials that support the findings of this study are available upon request.
